# Antibodies to Low-Copy Number RBC Alloantigen Convert a Tolerogenic Stimulus to an Immunogenic Stimulus in Mice

**DOI:** 10.3389/fimmu.2021.629608

**Published:** 2021-03-12

**Authors:** Arijita Jash, Chomkan Usaneerungrueng, Heather L. Howie, Annie Qiu, Chance John Luckey, James C. Zimring, Krystalyn E. Hudson

**Affiliations:** ^1^ Department of Pathology and Carter Immunology Center, University of Virginia, Charlottesville, VA, United States; ^2^ Bloodworks NW Research Institute, Seattle, WA, United States; ^3^ Department of Pathology and Cell Biology, Columbia University Irving Medical Center, New York, NY, United States

**Keywords:** alloantibody, red blood cell, alloimmunization, tolerance, antibody-mediated enhancement

## Abstract

Red blood cells expressing alloantigens are well known to be capable of inducing robust humoral alloantibody responses both in transfusion and pregnancy. However, the majority of transfusion recipients and pregnant women never make alloantibodies, even after repeat exposure to foreign RBCs. More recently, RBCs have been used as a cellular therapeutic—very much like transfusion, engineered RBCs are highly immunogenic in some cases but not others. In animal models of both transfusion and RBC based therapeutics, RBCs that do not induce an immune response also cause tolerance. Despite a robust phenomenology, the mechanisms of what regulates immunity vs. tolerance to RBCs remains unclear. However, it has been reported that copy number of alloantigens on the RBCs is a critical factor, with a very low copy number causing non-responsiveness (in both humans and mice) and also leading to tolerance in mice. Recently, we reported that an IgG2c specific for an RBC antigen can substantially enhance the humoral immune response upon transfusion of RBCs expressing that antigen. Herein, we report that an IgG2c converts RBCs with low antigen copy number from a tolerogenic to an immunogenic stimulus. These findings report the first known stimulus that induces humoral alloimmunization to a low copy number RBC alloantigen and identify a previously undescribed molecular switch that has the ability to affect responder vs. non-responder phenotypes of transfusion recipients.

## Introduction

Red blood cells (RBCs) have complex immunological properties that are only beginning to be unraveled. Considerable evidence has accumulated that circulating RBCs can regulate the innate immune system by multiple pathways (1). Moreover, exposure to foreign antigens on RBCs from members of the same species tends to be either tolerogenic, or weakly immunogenic in a self-limiting fashion. However, in some cases, alloantigens on RBCs can be highly immunogenic. Perhaps the strongest evidence of this dual capacity of RBCs is widespread epidemiologic data from the millions of humans who are transfused each year, which demonstrate that only 3-10% of transfusion recipients make detectable alloantibody responses. However, those who do make alloantibodies can make high titer IgG that cause hemolytic transfusion reactions and/or hemolytic disease of the fetus and newborn. The mechanistic underpinnings of why some transfusions cause immunity whereas most do not is poorly understood; however, there is evidence to support a combination of donor and recipient factors. Thus, RBCs have complex properties when they carry foreign proteins, representing a unique immunological stimulus.

In addition to the natural immunobiology of RBCs, considerable interest has been generated in recent decades regarding engineered RBCs as a cellular therapy. By using a variety of methods for “loading” RBCs with enzymes (either internally or on the surface), therapeutic approaches are being developed for anti-tumor therapy, inborn errors of metabolism, regulating coagulation, and managing toxicology (2). One of the problems with such therapies is the unwanted generation of immune responses to the therapeutic enzyme, requiring RBCs that do not elicit an immune response. Conversely, linking of microbial peptides or nanoparticles to RBCs have been used to demonstrate prototype vaccines with efficacy in animal models (3–5); in these cases, it is required that the RBCs are immunogenic. Finally, RBCs have been proposed as a therapeutic tolerogen themselves to treat autoimmune disease and/or other unwanted immunities (6–8). For the above reasons, understanding the factors that regulate if RBCs are immunogenic, non-immunogenic, or tolerogenic are critical.

One particular variable that is likely to be involved in regulating humoral immune responses to RBCs is the copy number of the antigen. In the case of RBC therapeutics, this can be purposefully controlled; however, in the case of transfusion this is determined by nature. As above, the epidemiology of alloimmunization in transfusion provides a panoply of human data. A wide variety of alloantigen copy number is found amongst blood groups. In some cases, the copy number varies widely from donor to donor, while the amino acid sequence of the antigen is unchanged. For example, RhD is the most immunogenic RBC antigens known in humans, inducing an alloantibody response in approximately 50% of RhD negative recipients. However, transfusion of RBCs with low copy number of RhD (e.g. weak D or Rh_DEL_) are generally considered to be non-immunogenic. However, even in these very low copy number cases, there have been rare reports of alloimmunization (9–11). Thus, a thorough understanding of how copy number affects immunological properties is important in both transfusion and RBC therapeutics.

In order to allow the study of RBC alloantigen copy number as an independent variable, we previously reported the generation of three different strains of transgenic mice that express the same human KEL glycoprotein of the K2 variant, but with different copy numbers on their RBCs (KEL_Hi_, KEL_MED_, and KEL_LO_) (12, 13). Analysis of immune responses to these donors has demonstrated that while RBCs from both KEL_Hi_ (14) and KEL_MED_ (15) induced an alloantibody response in wild-type recipients, K2_Lo_ RBCs induced no detectable IgG alloantibody response (12). Moreover, wild-type mice transfused with KEL_Lo_ were tolerized to the alloantigen, as they no longer made a detectable alloantibody response upon subsequent exposure to K2_Med_ with poly (I:C), a stimulus that reliably causes alloimmunization in naïve mice. Thus, as in humans, low copy number antigens appear generally non-immunogenic in mice. Nevertheless, given that some humans do make primary alloimmune response to low copy antigens, an essential question is what can make this happen?

Recently, we have reported that pre-treatment of mice with an IgG2c specific for an RBC antigen can substantially enhance the humoral immune response upon transfusion of RBCs expressing that antigen (16, 17). Herein, we tested antigen copy number as an independent variable with regards to how IgG2c affects immune response. We report that an IgG2c converts K2_Lo_ RBCs from a tolerogenic to an immunogenic stimulus. It is unclear if IgG2c can overcome tolerance previously established by transfusion with RBCs expressing a low copy number of antigen – this will be an important question in future investigations. Together, the findings in this report are the first known stimulus that induces humoral alloimmunization to a low copy number RBC alloantigen and identify a previously undescribed molecular switch that has the ability to affect responder vs. non-responder phenotypes of transfusion recipients.

## Material and Methods

### Mice

HOD (18), KEL_Hi_ (13), KEL_Med_ (13), and KEL_Lo_ (12) mice were generated as described in the referenced manuscripts. HOD mice were initially generated on an FVB background and subsequently backcrossed onto a C57BL/6J (B6) background for over 20 generations (HOD.B6). All K2 mice were created on a B6 background. Each of the KEL mice express the same variant of the human KEL glycoprotein that is K2+, Kp^b^+, Js^b^+. Wild-type C57BL/6J (B6 mice) were purchased from Jackson Labs (Bar Harbor ME). All recipient mice were female, 8–12 weeks of age, and were provided food and water ad libitum. Antibody and RBC infusions were given intravenously through lateral tail vein injection. All animal studies were carried out according to approved IACUC protocols from either the BloodworksNW Research Institute or University of Virginia.

### Generation, Expression, and Purification of Monoclonal Antibody Switch Variants

Specific clones were isolated and characterized from previously reported immunizations and myeloma fusions, using precisely the same methods (19). Two new clones are described herein, designated as PUMA3 and PUMA4. The heavy and light chain sequences of PUMA3 and PUMA4 were isolated, sequenced, and cloned into expression vectors to generate an IgG2c switch variant. Antibodies were expressed in CHO cells and purified to homogeneity by the same methods as previously described (19).

### Transfusions and Antibody Treatments

Antibodies were injected, RBCs were isolated, and transfusions were carried out as previously described (17, 20).

### Determining Post-Transfusion Circulation, Antibody Binding to Red Blood Cells, and Antigen-Modulation

RBCs from K2_Hi_,K2_Med_, and K2_Lo_ mice were labeled with DiO and control B6 or HOD RBCs were labeled with DiI as previously described (20). 50 microliters of each RBC type were mixed in a 1:1 ratio and transfused by lateral tail vein injection. Post-transfusion recoveries were determined by the ratio of K2+ RBCs to control B6 or HOD RBCs, which was itself normalized to the same ratio from the pre-transfusion specimen. This approach controls for variation in transfusion volume and phlebotomy. Direct antiglobulin tests were carried out by staining the RBCs with goat-anti-mouse Ig APC, and measuring APC signal after gating on DiO positive events (i.e. KEL RBCs). Indirect antiglobulin tests, which also measure antigen-modulation, were carried out by staining with PUMA3 followed by goat-anti-mouse Ig APC and using the same gating strategy (16, 17).

### Measuring Humoral Immune Responses to Red Blood Cells Alloantigens

Alloantibody responses were measured by indirect immunofluorescence using RBC targets by flow cytometry as previously described. Targets to measure anti-K included K2_Hi_ RBCs or human Kpa+Kpb- RBCs. Targets to measure anti-HOD were HOD RBCs. In each case, RBCs were incubated with the indicated amount of serum, washed, and then stained with a secondary antibody (goat anti-mouse Ig APC, SouthernBiotech). In all cases, control wild-type B6 RBCs were used to establish background signal. For each serum sample, antibody binding was quantified by subtracting the MFI on B6 RBCs from the MFI on antigen positive targets.

### Statistics

Statistical significance for longitudinal analysis of serum antibodies was determined by a repeated-measures analysis of variance (ANOVA) followed by a Dunnett multiple comparisons test against phosphate-buffered saline (PBS) control. For analysis of multiple groups at 1 time point, appropriate 2-way and 1-way ANOVA with a Dunnett multiple comparisons test was used. To indicate significance, ****p≤0.0001, ***p ≤0.001, **p≤0.01, *p≤0.05.

## Results

### Generation of Novel Monoclonal Antibodies Against Kp^b^ and a Common Kell Epitope

**Figure 1:**
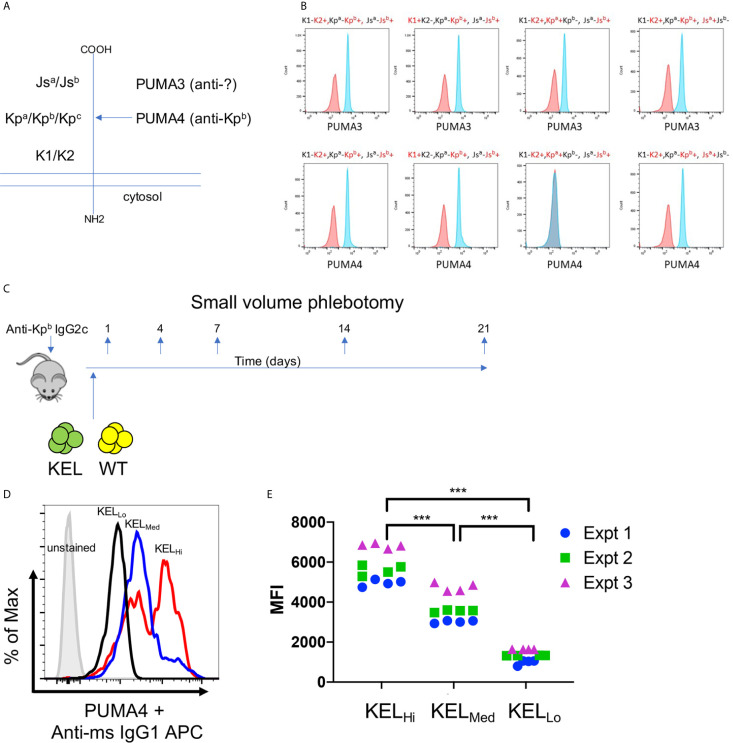
Generation of anti-KEL and anti-Kp^b^ monoclonal antibodies. **(A)** A diagram of the KELL glycoprotein and the location of the three major antithetical alloantigens is shown. **(B)** Human RBCs of the indicated phenotypes, chosen to isolate each major KELL antithetical alloantigen, were stained with either PUMA3 or PUMA4 antibodies (blue histograms). Staining was compared to negative control RBCs (red histograms) that were only treated with the secondary antibody. The same negative control histogram is used for each RBC phenotype with regards to testing PUMA3 or PUMA4 with those RBCs. **(C)** The general experimental design is shown as described in the main text. **(D)** RBCs from KEL_Hi_, KEL_Med_, or KEL_Lo_ were stained with PUMA4 followed by anti-mouse IgG1 APC and analyzed by flow cytometry. **(E)** RBCs from each donor mouse were stained with PUMA4 for each experiment performed and shown in **Figures 2** and **3**. Representative histograms of **(E)** are shown in **(D)**. Data were analyzed with a 2-way ANOVA with multiple comparisons test was utilized. ***p ≤ 0.001.

PUMA3 and PUMA4 are monoclonal antibodies that are first reported herein, but were isolated from a previously reported fusion of splenocytes from wild-type mice transfused with RBCs expressing the K1 variant of the human KEL glycoprotein (**Figure 1A**) **(** 19). PUMA3 and PUMA4 were tested with human RBCs expressing variants of the common antithetical human antigens in the Kell system (i.e. K1/K2, Kp^a^/Kp^b^ and Js^a^/Js^b^). PUMA3 was pan-reactive, indicating that it bound an epitope of the KEL glycoprotein that was not altered by K1/K2, Kp^a^/Kp^b^ or Js^a^/Js^b^ polymorphisms (**Figure 1B**). The epitope recognized by PUMA3 was not characterized further. PUMA4 was reactive with Kp^a^-,Kp^b^+ RBCs but not Kp^a^+Kp^b^- human RBCs; it was equally reactive with K1 vs. K2 and Js^a^ vs. Js^b^ RBCs (**Figure 1B**). Thus, the specificity of PUMA4 was determined to be anti-Kp^b^. We were unable to obtain Kp^c^+ RBCs, and thus have no information on PUMA4’s reactivity with Kp^c^. Both PUMA3 and PUMA4 are expressed by the isolated hybridomas as IgG1 antibodies. Because the hypothesis being tested is how RBC alloantigen copy number affects the effect of IgG2c on alloimmunization, PUMA4 was switched to IgG2c, as described in the materials and methods. PUMA3 and PUMA4 will be referred to as anti-KEL and anti-Kp^b^, respectively, throughout the rest of this manuscript. Except where indicated otherwise, the IgG2c form of anti-Kp^b^ is used.

The experimental design is shown in **Figure 1C**. Wild-type recipient mice were infused with anti-Kp^b^-IgG2c and then transfused with RBCs expressing the human Kell glycoprotein at different copy numbers [KEL_Hi_, KEL_MED_, or KEL_LO_]. Prior to transfusion, RBCs were labeled with a tracking dye (DiO) and were mixed with wild-type RBCs labeled with a separate dye (DiI); this is a well described approach that allows enumeration of surviving circulating RBCs by flow cytometry with normalization to circulating antigen-negative RBCs as an internal control (20). Peripheral blood was sampled at the indicated time points and tested for RBC survival, antibody binding (DAT), antigen modulation of KEL, and alloantibody formation—as detailed below.

Anti-Kp^b^ stained RBCs from KEL_Hi_, KEL_MED_, and KEL_LO_ donor mice in a pattern consistent with previous reports of antigen levels on RBCs from the tested strains stained with anti-K2, including the observation that KEL_Hi_ RBCs had a bimodal distribution of antigen, with both higher density and lower density populations (12, 13). These data confirmed that the Kp^b^ antigen was present at the predicted levels on each donor strain (**Figure 1D**). Antigen levels were tested for each donor mouse over three independent experiments to confirm consistency of antigen expression (**Figure 1E**).

### The Effect of Antigen Copy Number on Clearance and Antigen Modulation

**Figure 2:**
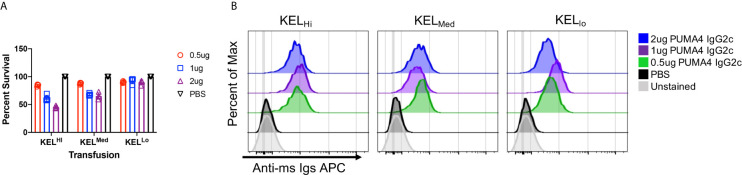
Differential clearance of RBCs based upon antigen copy number. **(A)** Twenty-four hour post-transfusion recoveries were determined (as described in *Materials and Methods*) for the indicated doses of antibody and for KEL_Hi_, KEL_Med_, or KEL_Lo_ RBCs. **(B)** The extent to which infused PUMA4 bound to the surface of transfused RBCs was determined by staining peripheral blood with anti-Ig APC, gating on the transgenic RBCs *via* DiO florescence, and then determining APC fluorescence on the gated population. This is a flow cytometry-based version of what is routinely referred to as the direct anti-globulin test. A representative experiment is shown, which was repeated 3 times with similar results. The experiment was repeated 3 times with similar results (representative shown); repeat experiments included in **Supplemental Figures 1** and** 2**.

Using the experimental design shown in **Figure 1C**, anti-Kp^b^ was titrated over three separate doses and each dose was tested with RBCs from either KEL_Hi_, K2KEL_Med_, or KEL_Lo_ donors. At 24 h post transfusion, all doses of anti-Kp^b^ caused clearance of K2_Hi_ and K2_Med_ RBCs in a dose dependent fashion (**Figure 2A**, repeat data shown in **Supplemental Figure 1**). In contrast, no significant clearance of KEL_Lo_ RBCs was observed. The binding of anti-IgG on the transfused transgenic RBCs (gating on DiO+ events) indicated that all doses of antibody bound to RBCs (**Figure 2B**, repeat data shown in **Supplemental Figure 2**). This was not due to background binding of anti-IgG binding to RBCs as no signal was detected control mice treated with PBS compared to unstained RBCs. Unexpectedly, the intensity of antibody binding to RBCs was similar at each antibody dose and for each donor strain.

**Figure 3:**
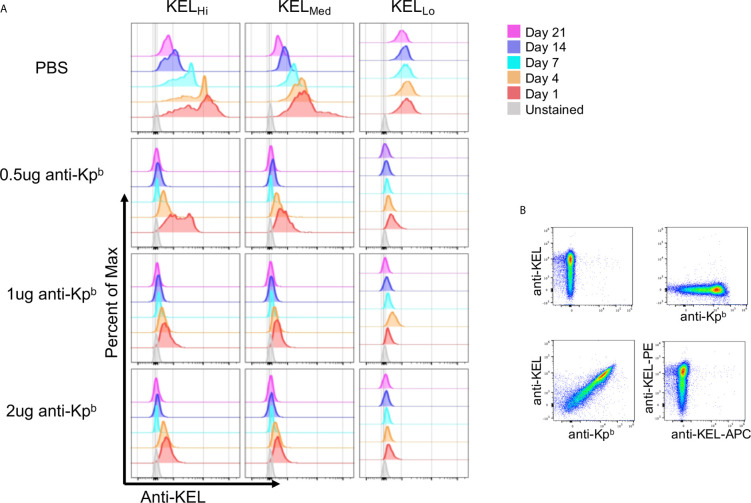
Antigen modulation as a function of antigen copy number. **(A)** Peripheral blood was obtained on the indicated days and the amount of KEL on the surface of RBCs was tested by staining with anti-KEL followed by anti-IgG1 APC, which recognizes anti-KEL but not the injected anti-Kp^b^. APC florescence was determined after gating on transfused RBCs *via* DiO. **(B)** KEL_Hi_ RBCs were stained with anti-KEL alone (upper left panel) anti-Kp^b^ alone (upper right panel), first with anti-Kp^b^ and then anti-KEL (lower left panel) or first with anti-KEL-PE followed by anti-KEL-APC (lower right panel). Prebinding of anti-Kp^b^ did not decrease binding of anti-KEL, excluding that antigen modulation was an artifact of antibody blocking. A representative experiment is shown, which was repeated 3 times with similar results. The experiment was repeated 3 times with similar results (representative shown); repeat experiments included in **Supplemental Figure 3**.

We have previously reported that binding of polyclonal anti-Kell causes antigen modulation of KEL epitopes on mouse RBCs (5). To assess this in the current setting, peripheral blood was stained with anti-KEL followed by fluorescently labeled anti-IgG1. Because the anti-Kp^b^ was IgG2c, then the anti-IgG1 would only detect anti-KEL. However, this approach would only give interpretable results if anti-Kp^b^ did not block binding of anti-KEL. To test this, KEL_Hi_ RBCs were stained with either anti-KEL followed by anti-IgG1 (conjugated to PE), anti-Kp^b^ followed by anti-IgG2c (conjugated to APC), or first incubated with anti-Kp^b^ then with anti-KEL and then with both secondary antibodies. This latter condition gave the same degree of staining for both antibodies as was observed for either antibody alone, indicating that binding of anti-Kp^b^ did not block subsequent binding of anti-KEL (**Figure 3B**). This was not because the assays could not detect blocking, in case it had occurred, since preincubation with anti-KEL IgG2c blocked binding of anti-KEL IgG1 (**Figure 3B**, bottom right panel). Thus, anti-KEL could be used to quantify the amount of KEL glycoprotein on the RBCs even when bound by anti-Kp^b^.

Peripheral blood was stained with anti-KEL followed by fluorescent anti-IgG1, and binding was assessed after gating on the transfused KEL RBCs (DiO+). At day 1 post transfusion, anti-KEL staining showed normal levels of expression on KEL_Hi_, KEL_Med,_ and KEL_Lo_ RBCs transfused into control mice that received only PBS and not anti-Kp^b^ (**Figure 3A**, repeat data shown in **Supplemental Figure 3**). However, all 3 doses of anti-Kp^b^ showed significant decrease in anti-KEL binding in both a time and dose dependent fashion. For example, 0.5 μg of anti-Kp^b^ caused about a 50% decrease in anti-KEL binding to KEL_Hi_ RBCs by 24 h post transfusion, which progressed to a significantly greater decrease by day 4 and to background levels (see unstained RBCs) by day 7—higher doses of anti-Kp^b^ showed the same trend but with faster kinetics. A similar overall pattern was seen with KEL_Med_ albeit with a lower starting point of antigen copy number. KEL_Lo_ had lost most anti-KEL binding by 24 h for all doses, with slightly less for the lowest dose (0.5 μg). It is worth noting that both KEL_Hi_ and KEL_Med_ RBCs had a progressive decrease in anti-KEL binding in control mice receiving PBS that started at day 7 and progressed through day 21. This is likely the result of an endogenous polyclonal antibody response to the KEL antigen generating antigen-modulation. Importantly, no decrease in anti-KEL staining was observed for KEL_Lo_ RBCs in control mice receiving only PBS, consistent with the previous report that KEL_Lo_ do not induce a detectable IgG humoral response.

### Anti-Kp^b^ IgG2c Enhances Humoral Alloimmunization to KEL_Hi_, KEL_MED_, and KEL_LO_

**Figure 4:**
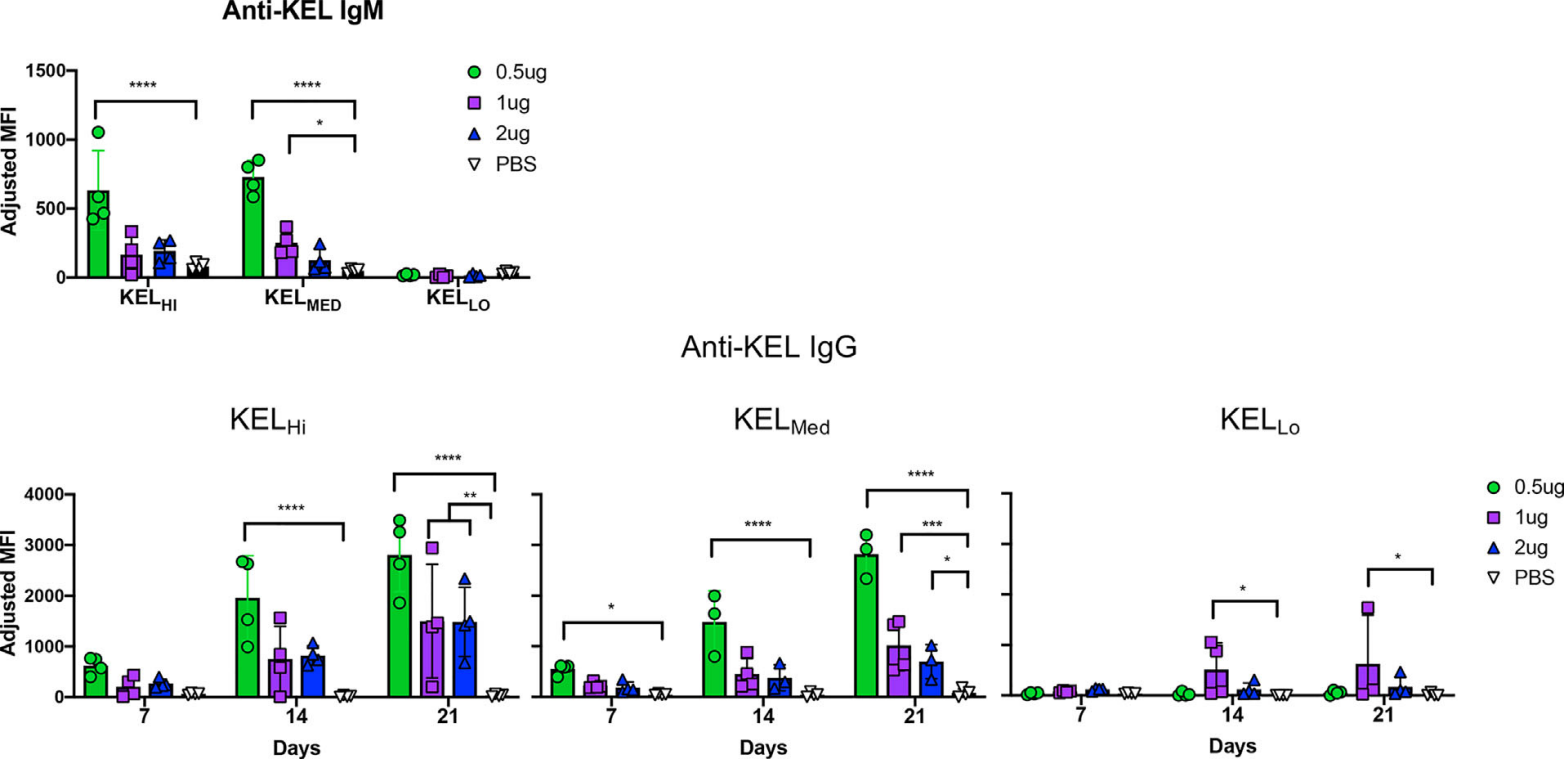
IgG2c enhances alloimmunization to KEL_Hi_, KEL_Med_, and KEL_Lo_ RBCs. Mice were treated as indicated in the experimental design (see **Figure 1C**) using the indicated dose of anti-Kpb IgG2c and the indicated RBC donors. Anti-KEL IgM and IgG were tested at the indicated times by incubating serum with KEL_Hi_ RBCs followed by anti-IgG. Background florescence was determined by staining wild-type RBCs and subtracted to calculate adjusted MFIs, as indicated in methods. The experiment was repeated 3 times with similar results (representative shown); repeat experiments included in **Supplemental Figure 4**. Data were analyzed with a repeated measures 2-way ANOVA with multiple comparisons test against PBS control. ****p ≤ 0.0001, ***p ≤ 0.001, **p ≤ 0.01, *p ≤ 0.05.

Serum collected from mice at days 7, 14, and 21 was assayed for generation of anti-KEL antibodies with a flow cytometry based crossmatch using KEL_Hi_ RBCs as targets. The lowest dose of anti-Kp^b^ caused a significant increase in IgM in response to both KEL_Hi_ and KEL_MED_, but not KEL_Lo_ RBCs (**Figure 4**, repeat data shown in **Supplemental Figure 4**). Higher doses of anti-Kp^b^ had a similar pattern, but only achieved statistical significance for 1 μg of anti-Kp^b^ with KEL_Med_. Surprisingly, anti-Kp^b^ caused a significant increase in anti-KEL IgG in response to RBCs from all 3 donor strains, including KEL_Lo_. By day 21, this enhancement achieved statistical significance for all doses of anti-Kp^b^ for K2_Hi_ and K2_Med_ but only for the 1 μg dose of anti-Kp^b^ for KEL_Lo_.

The ability of anti-Kp^b^ to induce a detectable IgG in response to KEL_Lo_ RBCs was particularly surprising, as we have previously reported that KEL_Lo_ does not induce an alloantibody response even when transfused in the presence of poly (I:C) (12). We have ruled out that this is simply a misinterpretation of detecting the anti-Kp^b^ that was injected and mistaking it for an endogenous antibody response by showing that the serology results are unaltered by using human RBCs targets that are Kpa+Kpb- and which do not detect anti-Kp^b^ (data not shown).

Because IgG injections can have general immunomodulatory effects separate from their antigen binding, additional studies were set up to test if anti-Kp^b^ was simulating alloimmunization without respect to its antigen specificity and if any IgG2c would have the same effect. This was tested by using a different RBC alloantigen to which anti-Kp^b^ does not bind, in this case the HOD alloantigen, which is a fusion protein of hen egg lysozyme (HEL), to the N-terminal half of ovalbumin (OVA), to the human Duffy blood group antigen (**H**EL-**O**VA-**D**UFFY = HOD) (18). PUMA6 IgG2c was also used, which is a monoclonal IgG that binds to the Duffy portion of the HOD antigen, referred to as anti-HOD hereafter.

**Figure 5:**
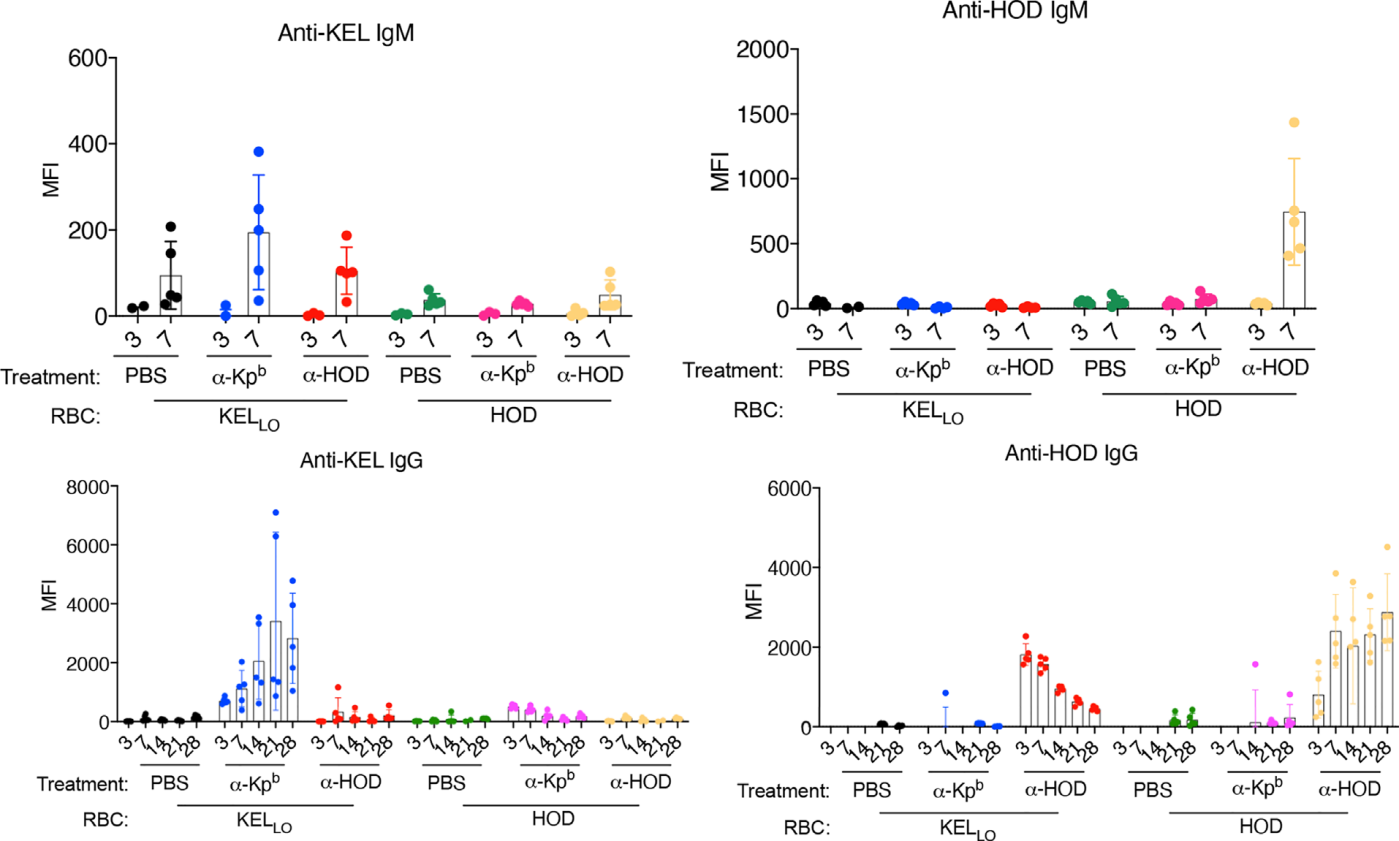
Enhancement of alloimmunization by IgG2c is antigen specific. Mice were treated as per the general experimental design (as in **Figure 1C**) with two different RBC donors (HOD or KEL_Lo_) and with two different antibodies (anti-Kp^b^ or anti-Fy). Serum was collected at the indicated days and tested for IgM or IgG against either HOD or KEL antigens using HOD or KEL_Hi_ RBCs as targets and using the appropriate isotype specific secondary antibodies (as in *Materials and Methods*). Each panel is labeled with regards to the RBC targets used, and therefore, the specificity of the antibody being tested. Treatments of each animal are listed below each panel. The experiment was repeated twice with similar results (representative shown); repeat experiment included in **Supplemental Figure 5**.

KEL_Lo_ RBCs or HOD RBCs were transfused into mice pretreated with either anti-Kp^b^ or anti-HOD (both IgG2c) (**Figure 5**, repeat data shown in **Supplemental Figure 5**). Serum was collected on days 3,7,14,21 and 28 for each group and tested for alloantibodies to KEL (using KEL_Hi_ targets) or against HOD (using HOD targets). Consistent with the above experiments, KEL_Lo_ RBCs did not induce a detectable IgG response alone, but had a significant anti-KEL IgG response when mice were pretreated with anti-Kp^b^. In contrast, no significant increase in anti-KEL was observed when KEL_Lo_ RBCs were infused into mice pretreated with anti-HOD. The anti-KEL antibodies were not due to detecting the anti-Kp^b^ that had been injected; the background signal for this can be observed in the group receiving anti-Kp^b^ and then HOD RBCs. Conversely, and consistent with previous reports, HOD RBCs induced only very low levels of IgG alone, but had a significant anti-HOD IgG response in mice pretreated with anti-HOD but not with anti-Kp^b^. The increased anti-HOD was not an artifact of detecting the anti-HOD that had been injected; that background can be observed in the mice receiving anti-HOD and KEL_Lo_ RBCs, and is consistent with previous reports that showed a strong signal at day 3 that then gradually decreased overtime (17). In contrast, mice receiving anti-HOD and HOD RBCs had a low signal at day 3 (presumably because anti-HOD was bound to transfused RBCs) and then the signal progressively increased overtime.

The failure of anti-Kp^b^ to enhance IgG responses to HOD RBCs rejects that anti-Kp^b^ is a general immune stimulator. The failure of anti-HOD to enhance anti-KEL in response to KEL_Lo_ RBCs rejects that anti-KEL responses are enhanced by IgG2c in general, regardless of its binding to RBCs. Together, these data demonstrate that anti-Kp^b^ induces an anti-KEL response to transfusion of KEL_Lo_ RBCs in an antigen-specific fashion that depends upon antigen binding activity.

## Discussion

Herein the binding of an alloantibody is identified as a factor that converts an otherwise low density tolerogenic stimulus to an immunogenic stimulus. This effect was antigen-specific, as anti-Kp^b^ did not enhance alloimmune responses to HOD RBCs nor did anti-HOD enhance alloimmunity to KEL_Lo_ RBCs. To the best of our knowledge, this is the first report to identify an immune stimulus that can cause alloimmunization to a low copy number RBC alloantigen, one that cannot be enhanced by traditional activators of innate immunity such as poly (I:C) (12).

The current studies also generalize the phenomenon of IgG2c enhancing immunogenicity of alloantigens on RBCs. Including this report, this has now repeatedly been observed with 5 different RBC donor strains (HOD, K1, KEL_Hi_, KEL_Med_, and KEL_Lo_) and three different monoclonal antibodies (anti-K1, anti-Kp^b^, and anti-HOD). The mechanisms by which IgG2c enhances alloimmunization is unclear; however we have reported that in the case of HOD and K1, Fc gamma receptors on dendritic cells are required (16, 17). Moreover, for the HOD system, IgG2c resulted in an increase in proliferation of HOD specific CD4+ T cells. Thus, it seems likely that opsonizing IgG2c is promoting increased antigen uptake by APCs and/or activation of APCs, both through FcγR ligation.

If increased antigen uptake is occurring, it is unclear if it is due to phagocytosis of whole RBCs or if antigen modulation of the RBCs results in isolated antigen complexes being consumed (21). In the current report, anti-Kp^b^ induced both RBC clearance and a decrease in detectable KEL antigen on KEL_Hi_ and KEL_Med_ RBCs. These finding of simultaneous clearance and antigen modulation are similar to what has been reported by Maier et al. with polyclonal anti-Kell using the KEL_Med_ system (22) and similar to what has been observed in humans for the KEL antigen in patients with autoimmune hemolytic anemia (23). In contrast, whereas antigen-modulation was obvious on KEL_Lo_ RBCs, only minimal clearance was observed. This observation for KEL_Lo_ RBCs seems to indicate that clearance of RBCs is not required for IgG2c mediated enhancement; however, given how few DCs are required to induce an immune response, and how many RBCs there are, it remains possible that the amount of consumption of RBCs required to increase an immune response is lower than what is detectable by enumerating circulating RBCs (as is suggested by other studies) (17).

It is unclear why a moderate dose of anti-Kpb (1 μg) but not a higher dose (2μg) had both a higher DAT and also the greatest enhancing effect for KEL^Lo.^ Likewise, higher doses of antibody had lower enhancement for KEL_HI_ and KEL_MED_. These were unanticipated findings, which raises the possibility that the stoichiometry of antigen-antibody binding complexes and cross-linking may be important (analogous to a prozone-like effect). Along these lines, the importance of cross-linking of RBC antigen has been reported with regards to antigen-modulation on RBCs (20, 24). The data in this manuscript are only correlative, and we cannot infer a causal link between clearance and/or antigen-modulation at the current time. Nevertheless, the data with KEL_Lo_ RBCs raise the possibility that increased antigen dose to APCs can occur, even in the absence of RBC clearance, if the antigen is removed from the RBCs (which still circulate).

The combined finding that in the case of KEL_Lo_ RBCs, IgG2c but not poly (I:C) enhanced alloimmunization is meaningful. Poly (I:C) signals through multiple pathways, including TLR3, RIG-MAVS, and PKR. Through these multiple pathways, poly (I:C) activates IRF3, IRF7, NF-kB and MAPK pathways. It has been reported that MAVS (and also the downstream induction of interferon-alpha) is required for enhancement of alloimmunization to RBCs in mice (25, 26), more strongly implicating IRF3. Consistent with this, poly I:C has no effect in IRF3/IRF7 double knockout mice (25). In contrast to poly (I:C), IgG2c signals through Fc gamma receptors (FcγR); we have recently reported that RBC bound IgG2c binds strongest to FcγRI and FcγRIV, with lower binding to FcγRIII and FcγRII. FcγRI, FcγRIII, and FcγRIV are activating FcγRs and each signal through the same common gamma chain (FcRγ), encoded by the FCER1G gene (27). Deletion of the FcRγ on dendritic cells prevents the enhancement of RBC alloimmunization by IgG2c (17). FcRγ activates the RAS-MAPK pathway, resulting in NF-kB activation and calcium mobilization. Thus, signal transduction in response to poly (I:C) and FcRγ have overlapping down-stream targets; however, a careful juxtaposition of the induced genes may be a fruitful avenue to elucidating what IgG2c is doing that allows alloimmunization to K2_Lo_.

The current findings have direct translational relevance to immune properties of human RBCs, with regards to both traditional transfusion and also RBC based cellular therapies. In the case of transfusion, antigen copy number per RBC varies widely amongst different human blood group antigens, from as low as 60 copies (i.e. type 17 weak D) to as high as 800,000 copies per RBC for glycophorin A, which carries the M and N antigens. Blood group antigens also differ in their immunogenicity. For protein antigens, RhD is the most immunogenic by far, followed by K>Jk^a^>Lua>E>c>M>C>Fy^a^>S (28). There is no general correlation between copy number and immunogenicity of blood groups. However, what is clear, is that when copy numbers of RBC alloantigens become extremely low, then immunogenicity decreases substantially. This is highly evident in the human RhD system. RhD has approximately 100,000 copies per red blood cell (RBC); however, there are a wide range of variant RhD phenotypes that have the same amino acid sequence as RhD but with substantially fewer copies; in particular, “weak-D” ranges from 60–5,200 copies depending on the weak D type. RhD is the most immunogenic RBC alloantigen, inducing a humoral immune response in as many as 50% of recipients. In contrast, alloimmunization of RhD-negative recipients to weak-D has only rarely been reported, and some have claimed it does not happen at all. To the best of our knowledge, the potential that human RBCs with low copy numbers of RhD tolerize people to RhD alloimmunization has not been tested.

The current studies lead to the hypothesis that IgG against RBCs is responsible for some humans becoming alloimmunized to weak-D RBCs. In this case, it would not be a pre-existing antibody to the RhD alloantigen, as this is what is being enhanced. Rather, it would be a low level alloantibody to a different antigen that was also expressed on the weak-D or DEL RBC. Such a mechanism would require cross-enhancement; however, we have recently reported that IgG2c cross enhances third party alloantigens not recognized by the alloantibody, so long as the antigen is co-expressed on the same RBC (17). One might argue that such an event would never occur, since we do not transfuse incompatible blood; however, that assertion is incorrect in two instances. First, cross-match incompatible RBCs are routinely transfused for “clinically insignificant” alloantigens. However, because an antibody does not cause hemolysis does not mean it doesn’t affect alloimmunization; indeed, in the current report, anti-Kp^b^ did not cause significant clearance of KEL_Lo_ RBCs but did enhance alloimmunization. Second, seminal studies done by Mollison et al. have demonstrated that the clearance of ^51^-Cr labeled RBCs in patients without detectable alloantibodies predicts the subsequent development of detectable alloantibodies – in other words there is a level of alloantibody that has a functional effect upon RBCs, but is below the level of detection in clinical assays. This is also a well-known phenomenon for patients who develop an alloantibody that then evanesces below the level of detection, and the patient is then transfused with RBCs expressing the antigen in question by a new medical service that has no record of the previous alloantibody.

With regards to RBC based cellular therapies, coating RBCs with IgG may be a highly effective adjuvant in the setting of RBC based vaccines. These findings may also be useful in instances where tolerance and/or non-responsiveness needs to be broken (e.g. tumor microenvironment). In contrast, when one is attempting to avoid immunity (e.g. enzyme loaded RBCs), ruling out IgG that are very low level, or that may be routinely ignored by the blood bank (i.e. “clinically insignificant alloantibodies) may be important to avoid immunization to the therapeutic RBCs. Similar issues may apply when using RBCs to induce tolerance, so as to avoid factors that oppose the therapeutic goal. In summary, and to our knowledge, the findings reported herein identify the first pathway that can induce alloimmunization to a very low copy number antigen on transfused RBCs. These findings have implications with regards to understanding alloimmunization to RBCs in humans, as well as immunobiology in general.

## Data Availability Statement

The raw data supporting the conclusions of this article will be made available by the authors, without undue reservation.

## Ethics Statement

The animal study was reviewed and approved by All protocols and experiments were carried out in accordance with relevant guidelines and regulations as approved by Institutional Animal Care and Use Committees (IACUC) at Bloodworks Northwest, University of Virginia, and Columbia University.

## Author Contributions

AJ, CU, JZ, and KH contributed to the conception and design of the study. All authors participated in the data acquisition and analysis. JZ and KH drafted the manuscript, and all authors provided critical revisions. All authors contributed to the article and approved the submitted version.

## Funding

These studies were supported in part by the National Institutes of Health, National Heart, Lung, and Blood Institute [P01HL132819 (JZ) and R01HL135248 (KH)].

## Conflict of Interest

The authors declare that the research was conducted in the absence of any commercial or financial relationships that could be construed as a potential conflict of interest.
