# Supplementary figures and images for: Antibodies to Low-Copy Number RBC Alloantigen Convert a Tolerogenic Stimulus to an Immunogenic Stimulus in Mice
Source: Front Immunol. 2021 Mar 12;12:629608. doi: 10.3389/fimmu.2021.629608 (PMC7994621; doi:10.3389/fimmu.2021.629608)

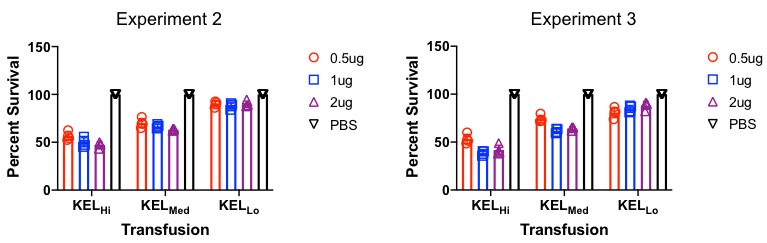

Supplement: Supplementary file 1 [file Image_1.jpeg]

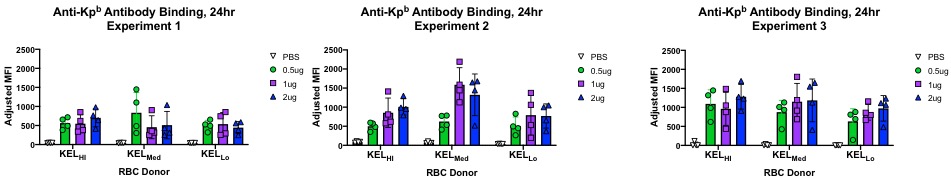

Supplement: Supplementary file 2 [file Image_2.jpeg]

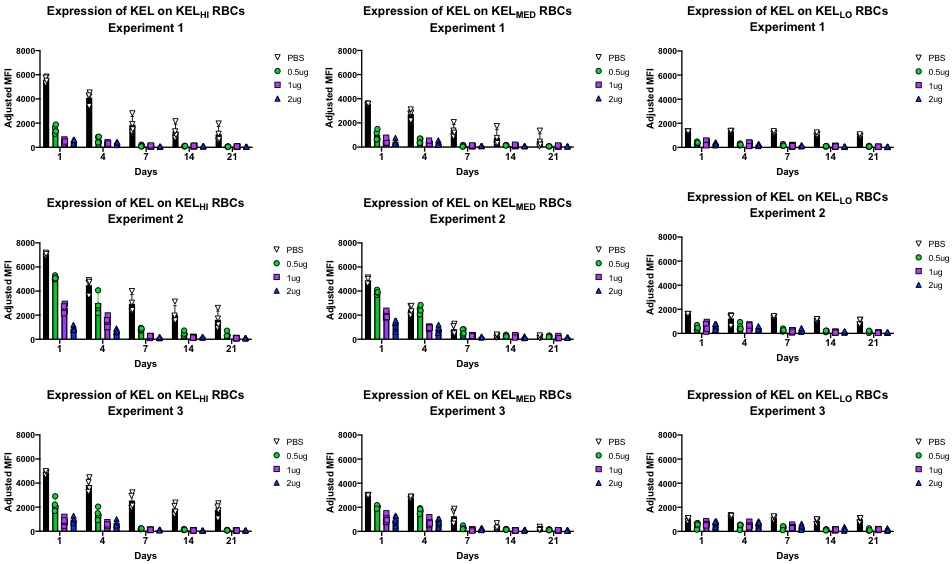

Supplement: Supplementary file 3 [file Image_3.jpeg]

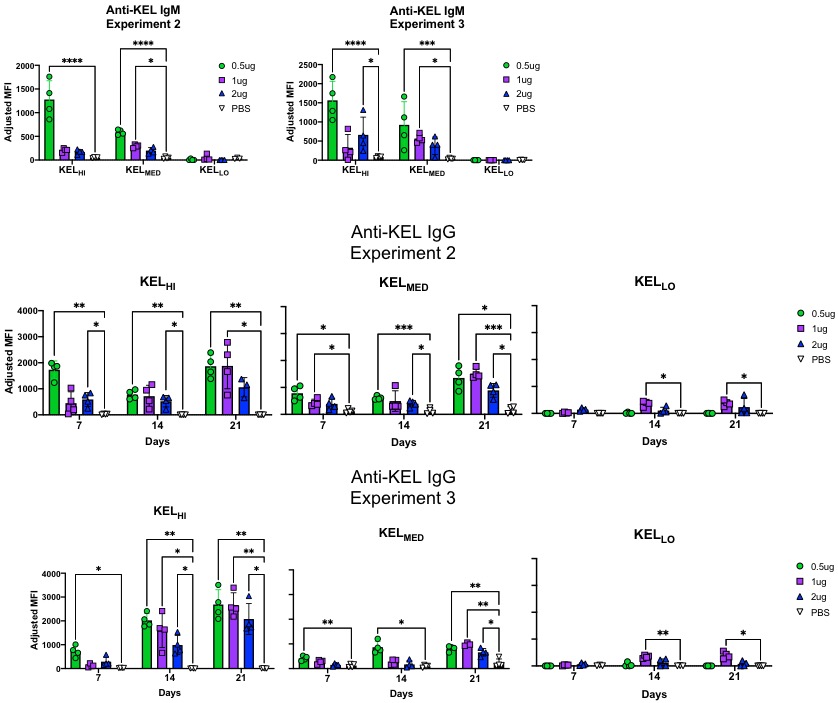

Supplement: Supplementary file 4 [file Image_4.jpeg]

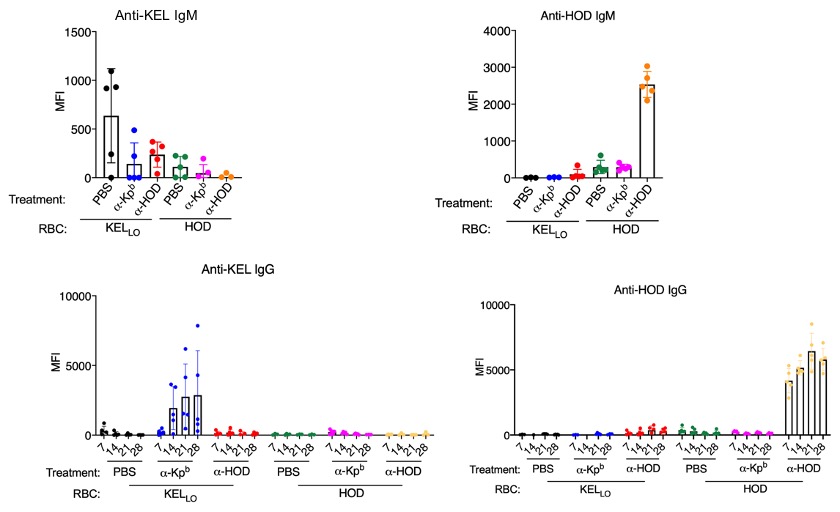

Supplement: Supplementary file 5 [file Image_5.jpeg]
